# Anatomy

**Published:** 1840-01-01

**Authors:** 


### ANATOMY.

I. The Surgical Anatomy of the Groin, the Femoral and Politeal Regions. By *Thomas Morton*, formerly one of the House Surgeons of University College Hospital. Illustrated with Lithographic Plates and Wood Engravings. London, Taylor and Walton, 8vo. pp. 207. 1839.

II. The Anatomist's Manual; or, a Treatise on the Manner of Preparing all the Parts of Anatomy, followed by a complete Description of these Parts. By *J. P. Maygrier*, M.D.P. Professor of Anatomy and Physiology; of Midwifery, and the Diseases of Women and Children; Physician to the Bureau de Charité of the Tenth Arrondissement; Member of the Medical Society of Emulation of Paris, of that of Practical Medicine in the same City; of the Medical Societies of Liège and Toulouse; of those of the Sciences in Maçon, Marseilles, &c. &c. &c. Translated from the last French Edition. London: George Henderson. 32mo. pp. 557. 1839.

III. The Surgical Anatomy of the Arteries, and Descriptive Anatomy of the Heart: together with the Physiology of the Circulation in Man and Inferior Animals. By *Valentine Flood*, A.M. M.D. T.C.D. Member of the Royal Irish Academy, Member of the Royal College of Surgeons in Ireland; and Lecturer on Anatomy and Surgery in the North London School of Medicine. London: S. Highley. 12mo. pp. 237. 1839.

IV. Text-Book of Anatomy for Junior Students. By *Alex. Jardine Lizars*, M.D. Fellow of the Royal College of Surgeons of Edinburgh, Lecturer on Anatomy, and Examiner to the University of St. Andrews. Part I. 12mo. pp. 272. 1839.

1. Mr. Morton informs us that the present publication is intended to form, with the Surgical Anatomy of the Perinæum already published, part of a Work, which, when it shall have been completed, will contain a description of the Surgical Anatomy of some of the most important regions in the human body.

"The drawings, from which the lithographic plates have been engraved, were very carefully made after my own dissections; as were also those from which the wood-engravings have been taken, with the exception of Nos. 4 and 5, which were accurately copied from similar plates in Hesselbach's Essay upon the Origin and Progress of Herniæ."

The execution of this work is highly creditable to Mr. Morton. So far as we have dipped into them, his descriptions of the anatomy of hernia are exceedingly correct. The work is well adapted for students, and is calculated to supply them with information of a very valuable character.

The plates are extremely well executed. There are eight coloured lithographs, besides several wood-cuts. We recommend the work strongly.

3. Dr. Flood informs us in his Preface:—

"My principal object in writing the following pages, has been to present the professional reader with an accurate description of the Heart and Arteries of the human subject, in as condensed a form as would be consistent with clearness. To make the work still farther available for practical purposes, I have added a description of the principal operations performed on the larger trunks, and incorporated a vast number of the varieties, which an operating surgeon should be prepared to meet in the course of his practice. In collecting the anomalies of the vascular system, I have freely availed myself of the labours of Farre, Baillie, Meckel, Haller, Barkow, Bouillaud, Otto, Gintrac, Burns, Barclay, Tiedemann, Geddings, Cruveilhier, Wardrop, and the authors of various articles, in the *Philosophical Transactions, Cyclopædia of Anatomy,* and periodical works of the present and several preceding years. I have also to express my special obligation to Dr. P. H. Green, who, since my settlement in London, has liberally per-permitted me to peruse his scarce and valuable work on the varieties of the arteries—a work of the deepest research, and abounding in the most accurate information."

"In the form of an introduction will be found an outline of the physiology of the circulating system, in man and inferior animals, illustrated by several wood-cuts."

As a sample of this work, which will be really a valuable acquisition to every operating surgeon we extract the following short passage. We confess it is almost like shewing a brick as a sample of a house.

"*Irregularities affecting the ligature of the common carotid artery.* A very remarkable case has been observed in a subject at the Richmond Hospital School, in which there was no common carotid on the right side—the external and internal carotid arising separately from the arch of the aorta. The order of the vessels were—right subclavian, right external carotid, right internal carotid, left common carotid, left subclavian. My colleague, Mr. Power, showed this preparation to the younger Tiedemann when he visited our School, and Tiedemann remarked that no similar case had been observed or heard of by himself or his father. Mr. Harrison observes in a note in his book on the Arteries, vol, i. p. 29 —'I have known two examples of the internal and external carotids arising on one side separately from the aorta;' but makes no other observation on the subject.

In some cases the common carotid is crossed in front by the inferior thyroid artery. In other cases the vertebral artery ascends behind it to pierce the third or second vertebra.* Cases are recorded, in which it ascended behind the angle of the lower jaw† before it bifurcated, and on the other hand, it may bifurcate as low as the inferior margin of the thyroid cartilage,‡ or at the sixth cervical vertebra§: lastly, it sometimes happens, that there is no bifurcation, the common carotid and internal carotid forming a continuous trunk, which gives off the branches of the external carotid.

There are references in other parts of this work to instances in which the common carotid gave off the inferior thyroid, superior laryngeal, pharyngeal ascendens, superior thyroid and right vertebral."

The only pity is that so useful a little work should be got up in so plain and unadorned a manner.

4. Our friend Mr. Lizars may explain himself.

"Having had ample opportunities of observing how much Students are generally occupied during lecture time in taking notes of the Anatomical Demonstrations which are then being delivered, I have prepared the present Treatise with the view of saving them that trouble. In taking this step I shall probably incur the disapprobation of those who imagine that it is advantageous for the

---

* "Burns p. 170. Hargrave, p. 69. Hird. L. Med. Gaz. Feb. 4, 1837."

† "Green says, it may divide as high up as 'the superior part of the styloid process,' which must mean at the base of the skull, but he gives no explanation, instance, or authority."

‡ "Hart. Todd's Cyclopædia Art. Carotid."

§ "Burns on the Heart, p. 285. Morgagni Epist. 29, Art. 20."

Pupil to be so engaged ; but this opinion, I feel convinced, is erroneous, and for the following reason—That the learner, when thus engaged, generally loses half the lecture, his eye and attention being necessarily removed from the object under description, upon which alone they ought steadily to be fixed. This little work will enable the Student to follow lectures on Anatomy with (I trust) every possible facility ; and will supply him, at the termination of his studies, when his time is invaluable, with the readiest means of reviewing this branch of Science within the narrowest limits. In the execution of my task I have steadily aimed at the greatest degree of conciseness compatible with the requisite minuteness, and with accuracy; and utility has been the great and constant object of my ambition."

And Mr. Lizars has succeeded à merveille.

---

A Lecture on Civilization; read at Northampton before the Members and Friends of the Society for the Diffusion of Religious and Useful Knowledge, on Monday, June 30, 1839. By *Archibald Robertson*, M.D. Published at the request of the Society.

Every one acquainted with the talents of Dr. Robertson would be prepared for an eloquent address on such a subject as the above—and they will not be disappointed. The lecture is not a medical one, and therefore our notice of this pamphlet must necessarily be very concise. The accomplished author arranges the causes of progressive civilization in two classes—divine and human. The first include language, laws, government, and religion :—the second comprises printing, intermixture of mankind by means of war and conquest—geographical discovery—commerce—colonization—and emigration—arts, sciences, literature, poetry and music. On all these topics Dr. Robertson makes some highly important remarks—with the exception of religion, which he properly leaves to his clerical colleagues, as falling more especially within their province. It cannot be expected that we should analyze Dr. Robertson's theory of the causes of civilization throughout its various ramifications. We were strongly tempted to make an extract from the " Art of Printing," as one main cause of mankind's improvement, and more especially as an art with which we have had something to do, for the last twenty or thirty years; but another subject, with which we have had some acquaintance, before these " piping times of peace" took place, called us off from leaden types to leaden bullets—from the press to the press-gang—from ink to blood—and from sheets of foolscap to the winding-sheets of our soldiers and sailors. The passage occurs under the head of war and conquest. It is *too* flattering to John Bull to be passed over.

" I have before hinted, that the principle here pointed out, may be usefully applied to the investigation of the varieties of National Character. Let us try what we can make of it by applying it, as a test, to that of our own country. England is confessedly the foremost nation of modern Europe; being, by common consent, considered at the head of the Civilization of the world. What has made her so ? Some will say ' her free institutions.' But I urge in reply, that to say so would be mistaking the effect for the cause; for, I contend, that it was the pre-existing superiority of her national character that enabled her to acquire those free Institutions that have so long been the birth-right of Britons, and the admiration of the world. My belief is, that the supremacy of the British People is owing to the frequent intermixture of races in the early stages of our national history. Most of you familiarly know, without being reminded by me, that the Ancient Britons, the primitive Celtic Inhabitants of our Island, were conquered by Julius Cæsar, about fifty years before Christ. The Romans kept possession of this Island for nearly five hundred years. Then came the

Saxons as Conquerors; next to them the Danes; and last of all the Normans. It thus appears that the blood that animates the stout heart of an Englishman is not one unbroken stream, but a fluid collected from many parent-fountains. Hence, the composite order of our national character; in which a nice observer might be at no loss to detect traces of the simplicity of the Celt, the greatness of soul of the old Roman, the solidity of the Saxon, the fire, with somewhat of the ferocity, of the Dane, and all the pride, and more than all the military ardour of the gallant Norman. This I take to be the true secret of that *Pluck* (to use a homely, but expressive, term) in which all true-born Englishmen especially pride themselves; that inherent courage which enabled our ancestors to wrest their free Institutions from the reluctant hands of the Plantagenets, and the Stuarts. What was it but good Old English *Pluck* that enabled our fellow-countrymen, of our own day and generation,—emulous of the deeds of their forefathers at Agincourt, and at Cressy,—to withstand the fury of an iron shower for eight or ten long hours, and at last to annihilate the overgrown military despotism of the modern Attila, on the plains of Waterloo? This was a deed of arms, such as the world has seldom seen; a deed of prowess, which, added to countless triumphs in every quarter of the globe, by sea and land, has exalted the bravery of Britons to a height of renown

'Above all Greek, above all Roman fame.'

Of course I do not ascribe *all* to the physical superiority of the race; moral causes have had their influence; and, amongst these, the public spirit arising out of our free institutions has not been the least conspicuous. Our pure reformed worship too has had its share; for religion is the strength of a nation, just as truly as 'sin is the reproach of any people.' Unless the national character had been sprinkled, and purified, by the living waters of the National Faith, neither the bullet nor the bayonet would have fulfilled the National hope, as weapons of defence. Climate also has had a marked effect. The climate of our native land, much as it is abused, has in it many redeeming qualities. It tends to make our numerous population hardy, enterprising, and free. Its very severity and caprice teach daily lessons of prudence, foresight, activity, and industry. It has been my lot, in the course of my life, to be familiar with various climes; and, I am inclined to give the preference to our own, with all its admitted uncertainty, over them all. I would say of the climate, what the delightful Poet Cowper, says of the Country itself:—

'England, with all thy faults, I love thee still!'

I firmly believe, on the evidence of Statistical Tables, that the English climate, on the whole, is as conducive to the duration of human life as any of the most favoured climates under the Sun. Advantages and inconveniences are distributed with a more impartial hand, than we, on a transient view, are apt to imagine. If the more Southern Climates are gilded with a brighter sunshine, perfumed with more fragrant gales, and decorated with a greater profusion of plants and flowers, they are, at the same time, perpetually exposed to pestilential heats, infested with noxious animals, torn by hurricanes, and rocked by earthquakes, unknown to the rougher regions of the North."

Our readers are well aware that these sentiments have been those which we have long preached—we mean those respecting climate. But when Dr. Robertson casts an eye over our own country, and deliberately reflects on the scenes which have too recently disgraced it in the eyes of Europe—such as the fanaticism of certain sects—the followers of Irving—Johanna Southcote,—the Kentish Christ, &c. not to mention the revivals of Scotland, the Chartist mania, and hundreds of other instances, of which the red Indian, the tawny Hindoo, and the jet black African would be ashamed, he will see that there is much need of the schoolmaster *at home*, as well as *abroad*, and that the society, of which he is a distinguished member, has a great deal to do, before they raise the lower orders of this country even to a level with those of many parts of the Continent—especially Holland and Prussia.

The sample which we have extracted from Dr. Robertson's pamphlet is by no means the best which we could have selected ; but it will, we are sure, induce a great many of our readers to peruse the whole brochure, which is equally creditable to the head and heart of its author.

---

Medical Report of the House of Recovery and Fever Hospital, Cork Street, Dublin, for Two Years, from 1st January, 1837; to 31st December, 1838. By *E. A. Kennedy,* M.D. President of the College of Physicians. 1839.

This Report appears to be drawn up with great care, and is calculated to give us an insight into those visitations of typhus which scourge but too frequently the sister Island. The reporter assures us :—

The extent to which fevers have frequently prevailed in Ireland has led to the establishment of hospitals for the special reception of poor persons suffering under that disease. It is stated in the Report of a Select Committee of the House of Commons, in the year 1830, that in 1817, on a moderate calculation, one million five hundred thousand persons suffered from fever in Ireland; of which number at least sixty-five thousand died.

The number of applicants for admission into the Fever Hospital for 1835 exceeded the average of some former years. The cases continued to increase in 1836, and communications were made by the government to the physicians of the Fever Hospital, and measures for augmenting the accommodation of applicants adopted. In a Report made by the physicians to the Government, we find the following passage :—

" It may, however, be stated, that the physicians have scarcely ever observed those circumstances which favour the progress of epidemic disease more strikingly exhibited than they are at present. They have frequently seen from ten to twenty individuals crowded into apartments of small dimensions, ill ventilated, filthy and offensive in the highest degree, the inmates in want of the necessaries of life, oftentimes without bed-covering, or even sufficient personal clothing."

And some can be found to wonder that the Irish are not quite satisfied. The starving and naked and famished victims of typhus should utter no sounds which may mar the mirth, or disturb the aristocratic quietism of their masters.

" In December, 1836, and for several months previous, typhoid fever predominated beyond all former observation. Among my own patients a great number of instances of unmixed typhus of the very worst description occurred, than I recollect to have seen for several previous years. The mortality also was much greater; and it is curious to observe, from a reference to my notes of the cases taken at this period, how, almost invariably, the tenth day appears to have been that on which the fatal termination of the disease took place.

In the conclusion of this month, or rather, I should say, early in January, 1837, an epidemic catarrh or influenza appeared, and prevailed so generally, that neither age, constitution, or class of individuals was exempted from the ravages of the disease; it prevailed, I think, more extensively, and the rate of mortality was greater in the higher classes of society."

" It has been questioned whether two epidemic diseases ever prevail contemporaneously in the same locality. The author can here only remark, that the one he has detailed was preceded, accompanied, and followed by typhus fever, which in intensity and in the numbers attacked, nearly equalled any which has been recorded in the history of this hospital. The same occurrence was observed in the epidemic catarrh which prevailed in Ireland in 1803, as recorded by Drs. Moriarty and Patterson."

When the influenza prevailed, the fever abated somewhat of its virulence, but, as the former declined, the latter increased. It began to get a-head in March, and attained its maximum in June.

The type of fever which characterized the present epidemic, and which prevailed so generally in 1836, was the pure typhus, or what has been, not unaptly, termed adynamic typhus : delirium, great prostration of the mental and muscular power, petechiæ, black sordes on the tongue, involuntary dejections, subsultus, and tendency to sloughing being frequently present.

There was sometimes a tendency to hæmorrhage, at other times to diarrhœa, and at one period there were many cases of erysipelas and diffuse inflammation.

The duration of the fever was protracted, and relapses were by no means so frequent as has been observed by the author during the prevalence of former epidemics. There was also another peculiarity in its attacking the young in an unusually large proportion. Thus, by a reference to the table, it will be found that so large a number as 1847, under the age of fifteen, were discharged during the year; and the author has never witnessed so many cases of genuine typhus, accompanied with extensive petechial eruption, as occurred even in those under the age of five years.

" At the commencement of the epidemic, well marked crisis was not observed; but during its progress, and more particularly in private practice, where opportunities were more favorable for observation, crisis by urine was frequent; and this was so repeatedly brought to my notice, that in many cases a favorable prognosis was not formed until a deposit in the urine took place, and which occurred generally about the 14th or 16th, and in some instances so late as the 20th day. I did not consider convalescence established if this did not occur; nor was it safe to allow nutriment to some patients who had a craving for food previous to its appearance. Frequently sleep, which I consider the best crisis in fever, supervened on the occurrence of the urinary deposit.

If the frequency of crisis was not observable, the universality of petechiæ was remarkable. This circumstance is not now looked on with so much apprehension by those conversant with the fever of this country."

" I have observed, however, if the eruption is coeval with the fever, (that is, occurs on the first day, in those cases which commence *uno ictu*) that it is not a favorable symptom. Again, where the eruption re-appears and becomes vivid at the termination of fever when it had previously declined, that it frequently denotes an unfavorable issue. The practitioner should not be misled by those cases where, having found them to decline on the chest and extremities, he thinks that they have re-appeared on an examination of the back; it is the position of the patient which has retarded the evanescence of this eruption in these cases.

The variety of the eruption observable during the greater part of the year was of the rubeolous description; while in the latter part of the year 1836, the purpura variety prevailed. These latter cases on recovery I think afford a greater immunity from future attacks of fever. In both however there is less danger of relapse than in fevers of ordinary type.

During the latter part of August I met with miliary eruption in two cases: one in a female of the name of Godby, in whom it was extensively diffused over the whole body; gastric symptoms were predominant in her case.

This variety of eruption was frequent with me in the year 1826; it was mostly confined to the chest, and accompanied by diaphoresis, which was the general critical termination of the fevers of that year."

We shall pursue the author through his running notice of symptoms, and select such observations or facts as seem to possess any practical importance.

*a.* In many cases of fever, and these the most severe, when delirium was present, the pupils were contracted; this, although a formidable symptom and leading the author to form an unfavorable prognosis, was yet by no means so dangerous a symptom as dilated pupils; the former occurred much oftener in those cases where there was great subsultus and picking of the bed-clothes.

The author may here remark that the contracted pupils oftener denote the paroxysm of mania than that of fever.

*b.* Error in diet was however not unfrequently the cause of violent delirium in the convalescent. In one case of a man named Bishop in the author's wards, an error of the nature alluded to caused a violent paroxysm of delirium, which rendered the issue of his case for many days extremely doubtful. And in the case of a gentleman mentioned to the author by his friend Mr. Wilmot, a similar irregularity occurred; delirium set in, and lasted for three months, during which period the patient made several attempts at self-destruction.

*c.* Deafness frequently occurred during the progress of fever; and in some convalescents remained a considerable time after their discharge from hospital; when a patient said, " I don't hear what you say, Sir," the sound was more gratifying than the expression, " I am better," the symptoms of improvement often not being so manifest in the latter as in the former case. At all times I considered deafness during fever in a more favorable light than acuteness of hearing.

*d.* Mania occurred in three cases—there were two instances of partial paralysis—epilepsy, apparently, in three cases.

" In some instances, epileptic convulsions are known to be suspended during an attack of fever: in confirmation of which the author begs to notice the following interesting case, which occurred to him several years since. The son of a tradesman, aged nine years, had been subject to epileptic fits from the age of two years, which latterly became more violent and frequent. During my attendance on him in severe typhus, with tendency to gangrenous sloughs, he had no return of the epileptic convulsions; and what is more remarkable that for seven years after the fever, during which period I had repeated opportunities of observing his case, he never had a renewal of an attack; and I think it more than probable that the febrile attack extinguished the previous disease."

*e.* The state of the pulse is recorded in three hundred and twenty-three cases. Although the author would by no means wish to supersede a careful observation of the pulse in fever; yet it must be remarked that the indications of the pulse in severe cases of typhus, are more uncertain and fallacious than in the milder cases, and more particularly in those of idiopathic fever. At an advanced stage of the more severe forms of the disease, a pulse of 120 denoted more danger than the same frequency at its commencement. In the case marked 63 in the tables, the pulse was intermittent all through the fever, while there was no such irregularity when convalescence was established. It is well known that intermission of the pulse often occurs in slight febrile attacks caused by a deranged state of the bowels.

In one case of a female in the tents, the pulse was only thirty while the respiration was thirty-seven.

Slowness of the pulse was frequently observed in an epidemic in this city noted by Dr. Rutty. It was remarked in most of those whose fever ended in a bilious purging, that the pulse from the beginning throughout the disorder was extremely slow and equal, not beating above forty times in a minute, which was not the case with those who had a diarrhœa at first.

*f.* To an experienced practitioner the tongue affords a more certain index as to the nature and progress of fever than any one symptom, or perhaps assemblage of symptoms; whether we regard its colour, shape, tremulous motion, or mode of protrusion among the great variety of its morbid appearances. To these remarks, however, the author might add numerous exceptions in the present epidemic; a few may be noted: a man named Johnson was admitted on the 11th day of fever, with a clean although somewhat tremulous tongue, and died on the 13th. Andrew Doyle, violent delirium; papular petechiæ; tongue clean; death. A female of the name of Murphy was considered to be improving on the 11th day of fever, as she was found to turn on her side; her tongue clean and moist, with intellect clear; when, on the 12th day, this improvement was suddenly put a stop to by the appearance of suffused eyes; fluttering pulse; and she sunk the same evening. These, and many others which might be adduced, will show the necessity of caution in forming our judgment from the apparently favourable appearance of the tongue, when the other symptoms do not correspond.

"In elderly persons (and I have witnessed typhus gravior in many individuals of an advanced age) the tongue was found to be fuliginous; and in one gentleman, subject to the gout, the tongue appeared as if washed with ink every morning, it was so black and moist; yet the dejections did not differ from the ordinary vitiated appearance of the secretions in typhus: on recovery, a length of time elapsed ere he regained his sense of taste.

Sometimes the tongue was found to be deeply indented with fissures, both during the progress of fever and in convalescence. Morbid taste existed for several days prior to the accession of fever in many individuals. The tongue remains white in many cases for a considerable time after convalescence, and these, by inattentive observers, have been reported either relapses from, or fresh cases of fever."

*g*. Gangrenous ulceration of the mouth occurred in several instances. In two, the disease was arrested by the application of the nitro-muriatic acid, by means of a hair pencil to the gangrened surface with camphorated spirit applied to the cheek externally; and the exhibition of wine with sulphate of quinine. In the present epidemic, as well as in former ones, the affection occurred in cases where no mercury had been given.

*h*. Parotid swellings were frequent, in one or two instances arising suddenly in one night; they did not appear to be critical, or attended with danger in adults: but when they occurred in children, particularly in scarlatina, when the exanthem was imperfectly developed, the appearance of these swellings was regarded as a most unfavourable symptom. Sometimes suppuration took place, which retarded convalescence.

*i*. Abscesses were not so frequently observed as they had been on former occasions. But the following cases are worth perusal:—

*Case* 1.—"A female, aged 30, was considered convalescent, when suddenly she was seized with coma and involuntary dejections; pupils dilated; her head having been shaved during the fever, a mustard cataplasm was applied to the scalp; leeches applied to the ears; and a terebinthinate enema administered. Thinking that sudden effusion on the brain had taken place, I did not calculate on finding her alive on the next day; but my surprise and gratification were extreme, when the nurse informed me that during the night a quantity of matter burst forth from the left ear, which still continued oozing on my visit. She left the hospital convalescent."

*Case* 2.—David Sibbald, a servant, æt. 15, was admitted on the 10th day of fever, with petechiæ; sordes on the tongue; delirium during the progress of the disease. An extensive slough formed on the sacrum, notwithstanding the most unremitting attention; indeed the tendency to gangrene was remarkable in his case, the scapular, dorsal, and iliac regions, even the legs exhibiting marks of discoloration; on the 16th day of his illness the pupils were observed to be dilated, and insensible to the stimulus of a strong light: this state continued for a week, at the expiration of which, suddenly a large quantity of pus issued from the ear, when some degree of consciousness returned, as he made an attempt to protrude the tongue when required to do so. The emaciation in his case was extreme; he left the hospital at the expiration of thirty-three days; several months elapsed, however, before he regained strength sufficient to undertake the most trifling duties of his situation.

*Case* 3.—A man, during a protracted fever, was suddenly attacked with symptoms of laryngitis; on the application of leeches and a blister on the throat, he thought himself relieved: but the relief was conjectured to have arisen from the discharge of an abscess in the vicinity of the glottis, as he threw up a quantity of matter. As the convalescence of fever became established, the laryngeal symptoms continued. Although on a careful examination of the chest, no disease of the lungs could be detected, yet, from the emaciated and feeble state of the patient, it was on consultation deemed unadvisable to perform tracheotomy with a view of relieving the laryngeal disease; mercury was therefore cautiously exhibited, but without any alleviation of the symptoms. He died on the end of the thirty-fifth day from his admission. On post-mortem examination the lungs were healthy, but an ulcer was found in the left rima glottidis.

*Case* 4.—" I was requested to assist a medical friend in the examination of an individual, who, he informed me, had died in consequence of pericarditis in combination with fever then prevalent. On opening the thorax a quantity of matter was found lying on the pericardium, which on careful examination was discovered to proceed from an abscess formed under the fascia of the neck, and which made its way behind the sternum into the mediastinum. Although this rare and very unusual occurrence may not have been the immediate cause of the disease, which in this case proved fatal, yet it will show the necessity of constant vigilance in fever, in order that we may be able to detect the formation of matter in situations where prompt measures are required to remove impending danger."

*j*. Pulmonary hæmorrhage was rife in May and June. This hæmorrhagic tendency, says our author, occurring at one period rather than at another, is important to note, as it points to a caution in the employment of the lancet, or more especially in the employment of leeches in individuals so circumstanced; for when it exists, it is difficult to arrest the hæmorrhage.

*k*. The author, on this as well as on former occasions, had taken considerable pains in endeavouring to ascertain the latent period of fever, which, from many causes, differs much in different individuals. In many instances the disease commenced as it were *uno ictu;* while, in the vast majority, a period elapsed from six days to eight weeks; the longer term being more frequently observed in the upper orders of society. From numerous facts presented to him, the author is of opinion that the cases in which the disease develops itself rapidly and with energy, are generally the most formidable and attended with danger: but the longer the latent period of fever, the more complicated the disease has appeared to him, and more difficult in the management.

*l*. The fever chiefly pervaded the lower classes of society. But a good many domestic servants were attacked. A hint to the rich to subscribe to fever hospitals, and, we may add, to a poor-law.

*m*. With respect to the practice pursued during the past year, the author seldom had occasion to employ general blood-letting; the application of leeches to the head, with tepid sponging; and cupping when the chest was engaged, having proved sufficient in numerous instances to moderate the symptoms. In some of the cases of diarrhœa, which occurred in the progress of fever and during convalescence, the application of a few leeches to the umbilical region was found beneficial. Blisters were applied where a tendency to coma appeared; they were injurious, however, in those cases where great irritability of fibre existed; and in many cases sent to hospital, when epispastics had been applied on the complaint of head-ache, their application proved a more positive injury than a large depletion. Opium was prescibed in the form of Dover's powder, in cases of typhus complicated with the different shades of delirium tremens; and in cases where great excitability existed with pervigilium for two or three nights in succession, a full anodyne was given, having premised depletion by the application of leeches to the temples. There is no remedy, in the author's opinion, which requires more discriminative observation than the employment of opium in the treatment of fever. Wine has been freely used during this epidemic; the average quantity prescribed by the author and his colleagues seldom exceeded eight ounces in the twenty-four hours. It has been justly remarked that wine is sometimes a better hypnotic than opium. An illustration of the type of fever is afforded by the quantity of wine consumed in one month compared with the corresponding month in 1838; thus, in June, 1837, the quantity of wine consumed was 4562 ounces; while in the same month, 1838, the quantity was only 1352 ounces.

*n.* "With respect to the pathology of this epidemic, the writer regrets that his researches do not enable him to throw any light upon the darkness in which the pathology of fever is involved. The only post-mortem appearance that was at all constant, was congestion of the vessels of the membranes of the brain. But not only was this not invariable, but it is so frequently found in every form of death where the agony is prolonged, that there can be no necessary connexion between it and the symptoms of typhus fever. In many cases it was accompanied by sub-arachnoid effusion, which was sometimes a limpid serous fluid; sometimes a layer of an opaque whitish gelatinous matter.

But if this epidemic typhus was not characterised by any uniform or constant pathological changes, it was very remarkable for the almost total absence of those abdominal lesions, which have been regarded by some as the invariable attendant of typhus fever. Indeed, in this respect, this epidemic affords a striking and conclusive refutation of the false and hasty generalizations of the French pathologists on this subject. In this respect also it differs altogether from the epidemic fever of 1826, (which the writer had an ample opportunity of investigating, while in charge of fever patients supported at the expense of the government at the Meath Hospital that year,) in which, in a large proportion of the post-mortem examinations made by him, more or less disease of the glands of Peyer was found."

*o.* Our Author appends a Table of Monthly Returns of the Admissions, Discharges, and Deaths of Fever Patients in the several Hospitals, Dublin: distinguishing the sexes; from 1st January to 31st December, 1837.

"This table exhibits a complete view of the rise, progress, decline, and intensity of the Fever of 1837 in this city.

It appears from it that, in that year, 11,085 fever patients were admitted into the Dublin Hospitals. Of these, 4,648 were admitted during April, May, June, and July, the period when the epidemic was at its height; and of the remaining 6,437, admitted in the eight other months, 1,030 were admitted in January, when the influenza prevailed.

The total number of deaths was 1,103, giving an average mortality of one in every $10\frac{1}{10}$.

The relative number of males and females attacked, as well as the relative mortality of the two sexes, as exhibited in this table, is very remarkable and well worthy of attention. The number of males admited to hospital was only 4,986; whereas of females there were 6,099. The total mortality of the males was 1 in every $8\frac{1}{8}$, while of the females it was only 1 in every $12\frac{1}{2}$; or,

In the males the mortality was .. .. $12\frac{1}{2}$ per cent.

In the females .. .. .. .. .. .. .. $8\frac{1}{3}$ do.

The relative mortality of the different hospitals, as shewn by this table, is a circumstance highly deserving attention, as well on account of the differences as of the agreements amongst them in this respect. Thus, while the mortality at the Hardwicke, Dun's, and the Meath hospital, was so high as 1 death in every seven: in Steevens's and the House of Recovery it did not exceed 1 in every 12.

There is no point in the history of fever respecting which we have more conflicting statements than this of the mortality in hospitals. It is usually supposed to depend on a great variety of circumstances, over which the physician has little or no control; such as, the extent of accommodation in proportion to the number; the facility or difficulty of admission into hospital; the ages to which admission is restricted; the character of the prevailing epidemic, and the period of its duration at which the observations are made. It seems, however, probable from the discrepancies as well as the coincidences in the rate of mortality in the Dublin Hospitals during the same epidemic, observed in the same periods of its progress, that this depends on fewer and more general causes than is commonly imagined. This view receives remarkable support from a consideration of the rate of mortality in the tents at the House of Recovery, Cork-street, as exhibited in the table. In these the patients were placed in apparently the same circumstances, and exposed to the same influences throughout the whole course of the epidemic; yet, nothing can be more variable than the mortality. Thus we find in May, 1 death only in every 12; in June, 1 in every 7; while in August, we have only 1 in every 19. That this did not depend on any variation in the nature of the epidemic itself appears in the highest degree probable, from a comparison of the rate of mortality in the tents and in the adjoining hospital. Thus in June, when the deaths in the tents amounted to 1 in every 7, the mortality in the house was so small as 1 in every 15.5."

And Dr. Kennedy adds:—

From an examination of these tables it appears that in this epidemic the greatest number of attacks occurred from the age of 10 to 15 years for the males, when the proportion is about 18 per cent.; and from the age of 15 to 20 for the females, when the proportion is nearly 16 per cent.

The number of females at every age previous to 70 exceeded the number of males.

| | |
|---|---|
| The number of females under 20 years of age is .. .. | 1,588 |
| And of males .. .. .. .. .. .. .. | 1,376 |
| Excess of females under 20 .. .. .. | 212 or |

a little more than 7 per cent.

In the Glasgow Fever Hospital, from October, 1835, to November, 1836, this excess was found to be above 8½ per cent.; and, as Dr. Cowan has observed, this fact should be kept in view, as it has an important bearing on the relative mortality of the sexes.

The number of both sexes from the age of 15 to that of 30 years was 2,628, or 42½ per cent.

The number under the age of 30 years was 4,517, or 73 per cent.

After the age of 45 the numbers rapidly diminished. Of the 6,724 cases computed in this table, 5,962 were under 45 years of age, and only 762 above it, being about 88 per cent. for the former, and 11 per cent. for the latter.

In viewing this table in reference to the *mortality* in fever, the first thing that strikes one is the great difference in this respect between the sexes: thus, while the total mortality of the males is 1 in 9½, that of the females is only 1 in 15.

At almost every period of life comprised in the table, the mortality of the males from fever exceeds that of the females. In the earliest period of life, up to 5 years, the difference in this respect is not striking. After this age it increases until at 20 the deaths amongst the males are 1 in every 16, while amongst the females they are only 1 in every 42. From 20 to 25 the numbers are again nearly equal; but from 25 to 40 the deaths are nearly twice as numerous amongst the males as the females, after which to 55 the difference becomes still greater—the proportion for the males being 1 in 3, for the females nearly 1 in 11. From this time to the 80th year the mortality is nearly the same in both.

The rate of mortality is greatest both in males and females at 60.

Looking to the total number of deaths at different ages, it would appear that the mortality in fever is considerable up to 5 years of age, from which to 15 it is remarkably small, after which it increases, and goes on almost steadily augmenting up to 60.

We have taken out most of the important passages from this Report, and presented them to our readers. Of the Report itself we would speak in high terms. It forms a valuable pendant to that of Dr. Cowan, of Glasgow, and deserves, like *it*, to be studied. The subject of epidemic typhus begins to claim serious attention, even in London, and the body of practitioners should be in possession of the facts connected with it.

